# 1-C Metabolism—Serine, Glycine, Folates—In Acute Myeloid Leukemia

**DOI:** 10.3390/ph14030190

**Published:** 2021-02-26

**Authors:** Kanwal Mahmood, Ashkan Emadi

**Affiliations:** 1 Department of Medicine, School of Medicine, University of Maryland, Baltimore, MD 21201, USA; kanwal.mahmood@som.umaryland.edu; 2 Marlene and Stewart Greenebaum Comprehensive Cancer Center, University of Maryland, Baltimore, MD 21201, USA; 3 Department of Pharmacology, School of Medicine, University of Maryland, Baltimore, MD 21201, USA

**Keywords:** amino acid metabolism, cancer therapy, leukemia, amino-acid-degrading enzymes, amino acid restriction in cancer

## Abstract

Metabolic reprogramming contributes to tumor development and introduces metabolic liabilities that can be exploited to treat cancer. Studies in hematological malignancies have shown alterations in fatty acid, folate, and amino acid metabolism pathways in cancer cells. One-carbon (1-C) metabolism is essential for numerous cancer cell functions, including protein and nucleic acid synthesis and maintaining cellular redox balance, and inhibition of the 1-C pathway has yielded several highly active drugs, such as methotrexate and 5-FU. Glutamine depletion has also emerged as a therapeutic approach for cancers that have demonstrated dependence on glutamine for survival. Recent studies have shown that in response to glutamine deprivation leukemia cells upregulate key enzymes in the serine biosynthesis pathway, suggesting that serine upregulation may be a targetable compensatory mechanism. These new findings may provide opportunities for novel cancer treatments.

## 1. Introduction

Acute Myeloid Leukemia (AML) is a heterogeneous hematologic neoplasm characterized by clonal evolution of hematopoietic stem/progenitor cells resulting in disruption of normal blood cell production and function. Despite the new approvals of therapeutic agents as well as allogeneic stem cell transplantation, the five year survival rate for patients with AML remains less than 40% [1], underscoring the need for novel therapeutic strategies such as targeting metabolic pathways in AML that have recently become better understood. 

## 2. Metabolic Dysfunction in AML

Metabolic alteration, in which malignant cells alter metabolism by pushing the cell towards more glycolysis/glutaminolysis and dysfunctional oxidative phosphorylation, is now considered a hallmark of cancer [2,3]. Studies in AML have shown alterations in glucose (Warburg effect), fatty acid, folate, and amino acid metabolism pathways [2,4]. Due to metabolic reprograming, neoplastic cells are able to proliferate and grow, and these metabolic liabilities can be exploited for the treatment of AML. Folate metabolism supports a set of biochemical reactions known as one-carbon (1-C) metabolism, which is a universal metabolic process that serves to produce and transfer 1-C units for biosynthetic processes in the cell including nucleic acid synthesis [5]. 1-C metabolism has been shown to be frequently altered in hematologic and solid neoplasms [4]. Anti-folates have been among the first efficacious anticancer agents and still hold a significant role in the treatment of many hematologic and solid malignancies. This review will discuss the current state of targeting 1-C metabolism in AML as well as highlight emerging therapeutic approaches, with a focus on novel combination therapies that also target glutamine metabolism. 

## 3. De Novo Serine/Glycine Biosynthesis

### 3.1. Serine

Serine is an amino acid that is synthesized through the de novo serine biosynthesis pathway and provides precursors to produce purines, pyrimidines, lipids, and antioxidants [6,7]. Serine biosynthesis begins with the conversion of glycolysis intermediate 3-phosphoglycerate (3-PG) to 3-phosphohydroxypyruvate (3-PHP) by the phosphoglycerate dehydrogenase (PHGDH) enzyme. Conversion of glutamate to α-ketoglutarate mediated by phosphoserine aminotransferase (PSAT1) results in amination of 3-PHP producing 3-phosphoserine (3-PS). The final step involves the hydrolyzation of 3-PS to serine by phosphoserine phosphatase (PSP). The three enzymes involved in the serine biosynthesis pathway are reported to be upregulated in different neoplastic cells [8,9,10]. Specifically, it was demonstrated that silencing PHGDH has a detrimental effect on leukemia cell growth and survival [11,12] and PHGDH gene overexpression was among the 4-gene signature reported to be an independent negative prognostic marker in patients with AML [10].

### 3.2. Glycine

Glycine synthesis is an important reaction in which serine is used as a substrate. Serine hydroxymethyltransferases (SHMT), cytosolic (SHMT1) and mitochondrial (SHMT2), are responsible for the conversion of serine to glycine [13]. Glycine is one of the major sources of carbon donation for pyrimidine and purine biosynthesis involving the folic acid cycle [4,14,15]. Dependency of neoplastic cells on serine/glycine has been reported and can be further exploited pharmacologically [4,6]. For example, as a proof of concept a dramatic decrease in colon cancer growth was reported following dietary restriction of serine and glycine [8,14]; a strategy that may also be applied to various hematologic neoplasms.

Additionally, serine and glycine are heavily involved in the maintenance of cellular oxidative homeostasis [7,16]. Glutathione (GSH) is a tripeptide that consists of the amino acids cysteine, glycine, and glutamate [16]. GSH is the most abundant metabolite in the cell and is an important antioxidant that prevents oxidative damage caused by reactive oxygen species (ROS) and maintains the appropriate ratio of Nicotinamide adenine dinucleotide phosphate oxidase/Nicotinamide adenine dinucleotide phosphate (NADPH/NADP) [4,16]. Downregulation of any key enzymes in the serine biosynthesis pathway causes a decrease in GSH expression and subsequent increase in ROS production [6,11]. 

## 4. 1-C Metabolism 

### 4.1. Folate Cycle

Folates are important for cellular metabolism, and outputs of the folate cycle include components that are essential for the synthesis of many macromolecules [8]. Studies by have shown that in mammalian cells most of the 1-C units used in folate metabolism are derived from serine catabolism in mitochondria, [17] allowing the conversion of tetrahydrofolate (THF) to 5,10-methylenetetrahydrofolate (CH2-THF) by SHMT2. Folic acid can also be enzymatically reduced to dihydrofolate (DHF) and then further catalyzed by dihydrofolate reductase (DHFR) to produce THF [4]. CH2-THF is reduced to 5-methyl THF (CH3-THF) by the enzyme CH2-THF reductase (MTHFR) [4,6]. The concluding step of the folate cycle is the demethylation of CH3-THF complex, back to THF through the transfer of the methyl group to vitamin B12 [18]. This final step of the folate pathway is linked to the start of the methionine pathway, as the methyl group bound to vitamin B12 and methionine synthase (MS) is transferred to homocysteine, converting it to methionine [18]. Since the folate cycle is coupled with the methionine cycle, it is therefore essential for producing methionine and homocysteine.

CH2-THF dehydrogenase 2 (MTHFD2), an NAD+-dependent enzyme that is indirectly involved in 1-C metabolism, has been shown to be the most differentially expressed metabolic enzyme in cancer compared with normal cells [19]. Pikman et al. [20] reported that the suppression of MTHFD2 by shRNA impaired growth and promoted differentiation in AML cell lines. Furthermore, they showed that MTHFD2 suppression decreased leukemia burden and prolonged survival in MLL-AF9 mouse leukemia models and a human xenograft model [20].

Polymorphisms in the gene coding region for DHFR have been implicated in chemoresistance to the anti-metabolite methotrexate in acute lymphoblastic leukemia (ALL) [4,21]. Studies by Dulucq et al. [22] showed that a single nucleotide polymorphism (SNP) in the promoter region of DHFR at A317G results in higher transcriptional activity of this enzyme, thereby conferring resistance to methotrexate treatment. These findings underscore the need for novel therapeutics that can bypass polymorphism-associated chemoresistance.

### 4.2. Methionine Cycle

The methionine cycle is the second half of the 1-C metabolism pathway. It is directly involved in the production of GSH, methylation of proteins, methylation of nucleic acids and subsequent epigenetic modulation, as well as production of universal methyl group donor S-adenosylmethionine (SAM) [4]. The cycle begins with the demethylation of CH3-THF and conversion of homocysteine to methionine, which is subsequently converted to SAM by methionine adenosyltransferase (MAT). SAM is demethylated to produce S-adenosylhomocysteine (SAH), which is deadenylated to form homocysteine, completing a full turn of the methionine cycle [23]. Reduction of homocysteine to cysteine along with covalent bindings to glycine and glutamate produces GSH [4,5,6]. 

In a study by Barve et al. [24] deprivation of exogenous methionine disrupted methionine and SAM metabolism, resulting in significant apoptosis and global changes in cellular methylation in AML cells. Furthermore, pharmacologic inhibition of SAH by deazaadenosine resulted in a drastic prolongation of overall survival of MLL-R xenograft mouse model of AML.

Genetic polymorphisms in the 1-C pathway have been studied extensively and are associated with numerous conditions, including cancer. The most well-studied polymorphism is the c.677C>T in the coding region of the MTHFR gene [25]. This non-synonymous polymorphism encodes a valine to alanine substitution on residue 222 [26], resulting in overexpression of both folate and homocysteine. Recent studies have shown that elevated homocysteine levels are a risk factor for diseases such as Alzheimer’s and cancer [27], highlighting the significance of polymorphisms as a variable in combination treatment. 

While targeting serine/glycine/methionine has shown to be promising in the pre-clinical models of AML, these metabolic vulnerabilities should be combined with other clinically relevant amino acid-focused strategies to be translated efficiently and in a timely manner for prime-time clinical use. We propose that interference with glutamine metabolism is one of such promising strategies that is already utilized at the patient’s bedside for treatment of leukemias, lymphomas, and some solid tumors [28,29,30,31].

## 5. Glutamine Metabolism

Intracellularly, glutamine is the most abundant amino acid with tissue concentrations of approximately 20 mM with the highest concentration in skeletal muscle [32,33]. It has also the highest concentration among all amino acids in human plasma, with concentrations ranging from 300 to 900 µM [33]. Multifaceted utilization of glutamine includes: (1) contribution to TCA intermediates and ATP synthesis via a process called glutaminolysis [4], (2) contribution to nucleotide synthesis as a nitrogen donor, (3) maintaining redox homeostasis by contribution to the formation of glutathione [11], (4) contribution to fatty acid biosynthesis via cytoplasmic reductive carboxylation mediated by the enzyme isocitrate dehydrogenase 1 (IDH1), and (5) participation in the regulation of the mechanistic target of rapamycin (mTOR) complex 1 (mTORC1) as the master cell proliferation checkpoint via leucine transport mechanism [34,35]. Transportation of glutamine from the extracellular compartment into the cell is facilitated through specific families of solute carrier type (SLC) transporters that are found on the cellular and mitochondrial membranes which include SLC1, SLC6, SLC7, and SLC38 [36].

### Glutamine Metabolism and Cancer 

Hematologic and solid malignancies have both demonstrated a crucial dependence on glutamine for survival and proliferation; hence, interfering with glutamine metabolism and its plasma supply have emerged as promising clinically relevant therapeutic approaches for cancer [11]. Neoplastic cells frequently upregulate glutamine transporters [37] in response to their increased demand for energy, nucleic acid synthesis, and need to balance cellular oxidative state [1,37]. Conversion of glutamine to glutamate and ammonia by the glutaminase enzymes is the first step of glutaminolysis, which then feeds the mitochondrial Krebs cycle – even in the limited supply of glucose [38]—to provide energy to rapidly dividing neoplastic cells. A particular isoform of glutaminases known as glutaminase-C (GAC) is overexpressed in AML cells. Studies by Jacque et al. [39] showed that not only did pharmacologic inhibition of GAC decrease cellular proliferation and survival of AML cells in vitro and in vivo, but it also had a negative influence on mitochondrial respiration. Importantly, normal CD34+ hematopoietic cells were not affected by GAC inhibition. 

There are four major methods to target glutamine metabolism for treatment of different cancers. These strategies include, (1) depletion/decreasing plasma glutamine by clinically available FDA-approved asparaginase products [29,30,40], (2) inhibition of the glutaminase enzymes by different small molecule inhibitors currently being tested in clinical trials [41,42], (3) interference of glutamine transportation by small molecule inhibitors of SLCs [43,44], and (4) deceiving the neoplastic cells with glutamine antagonists/analogs [45]. Here, we propose that exploiting glutamine metabolism combined with the inhibition of 1-C metabolism has the potential to be efficacious for several cancers, particularly AML.

## 6. Glutamine Contribution to 1-C Metabolism

### 6.1. Glutamine, Serine, and 1-C Interaction 

**Figure 1:**
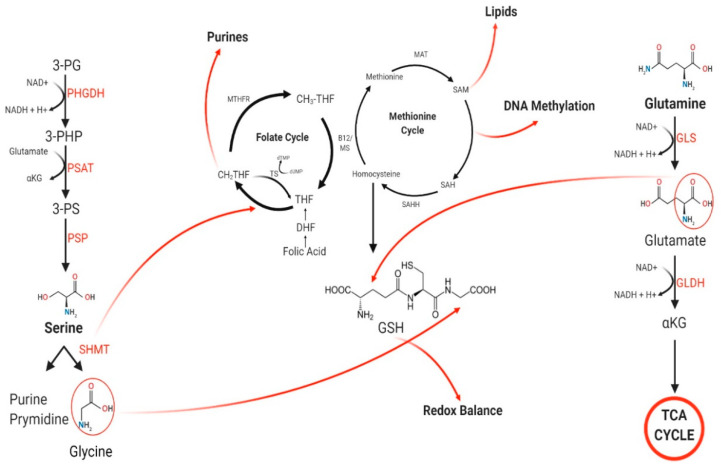
Schematic of the interaction between the three metabolic pathways (serine biosynthesis, 1-C metabolism, and glutamine metabolism). Serine is synthesized from 3-phosphoglycerate (3-PG) by phosphoglycerate dehydrogenase (PHGDH), phosphoserine aminotransferase (PSAT), and phosphoserine phosphatase (PSP). Serine is further converted to glycine by SHMT, which donates a carbon to the folate cycle. Tetrahydrofolate (THF) is converted to CH3THF by TS and MTHFR to complete the folate cycle. During demethylation of CH3THF, a carbon is donated to the methionine cycle by Vitamin B12. Methionine is synthesized to homocysteine by methionine adenosyltransferase (MAT) and SAAH, producing S-adenosylmethionine (SAM) and S-adenosylhomocysteine (SAH). Glutamine is converted to glutamate by the enzyme glutaminase. The side chain of glutamate is then used to form GSH. Abbreviations 3-PG—3-phosphoglycerate; PHGDH— phosphoglycerate dehydrogenase; PSAT—phosphoserine aminotransferase; 3-PHP—3-phosphohydroxypyruvate; 3-PS—3-phosphoserine; PSP—phosphoserine phosphatase; SHMT—serine hydroxymethyltransferase; TS—Thymidylate Synthase; dUMP—deoxyuridine monophosphate; dTMP—deoxythymidine monophosphate; MTHFR—methylenetetrahydrofolate reductase; THF—tetrahydrofolate; CH3THF—5-methyltetrahydrofolate; CH2THF—5,10-methylene-THF; B12—Vitamin B12; MS—Methionine synthase; MAT—Methionine adenyltransferase; SAM—S-adenosylmethionine; SAH—S-adenosylhomocysteine; SAHH—S-adenosylhomocysteine hydrolase; GSH—Glutathione; GLS—Glutaminase; GLDH—Glutamate dehydrogenase; TCA—tricarboxylic acid. Created with BioRender.com (accessed on 16 February 2021).

In order to efficiently target these metabolic pathways, it is important to understand the mechanisms which allow them to interact with one another. Serine biosynthesis directly interacts with the folate cycle by donating a carbon from the serine side chain. The folate cycle then directly interacts with the methionine cycle during the demethylation of CH3-THF to THF, and lastly glutamate derived by glutamine directly interacts with the homocysteine cycle during the production of glutathione (Figure 1). Studies by Polet et al. [11] have observed an increase in key enzymes involved in the serine biosynthesis pathway as well as a decrease in GSH following endogenous glutamine depletion. The interaction between serine and glutamine seems to be indirect rather than direct; however, the exact mechanism of interaction between glutamine and serine is yet to be determined. 

### 6.2. TP73 

A potential route that bridges the interaction between serine and glutamine could be through the TP73 transcription factor. The TP73 gene maps to chromosome 1p36.33, and it is a part of the TP53 family. p73 is a homolog to p53, and has two protein isoforms, TAp73 and ΔNp73 generated by alternative splicing and alternative promoter usage [46]. TAp73 induces both cell cycle arrest and apoptosis, whereas ΔNp73 inhibits both TAp73 and p53-induced apoptosis. Transcriptionally, TAp73 controls glutaminase-2 (GLS-2) and favors the conversion of glutamine to glutamate [47,48]. Glutamate then drives the serine pathway [49] by transferring an amino group 3-PHP by the enzyme PSAT, which yields 3-PS and α-KG. Interestingly, both Tp73 isoforms have shown to be highly expressed in neoplastic cells and overexpression of these isoforms results in tumor growth. Furthermore, TAp73 is highly expressed in AML cells that harbor recurring genetic abnormalities such PML-RARA, RUNX1-RUNX1T1 and CBFB-MYH11 [49,50]. Clinically, Lucena-Araujo et al. [51] have shown patients with AML that had a high ΔNp73/TAp73 ratio had a lower survival rate when compared to patients with a low ΔNp73/TAp73 ratio (high expression, ≥1.6; low expression, <1.6). This indicates that Tp73 significantly contributes to the growth and proliferation in AML cells. These results suggest that TAp73 promotes the serine biosynthesis pathway through transcriptional upregulation of the GLS-2 and the subsequent conversion of glutamine to glutamate in AML, and that the inhibition of TAp73 can potentially be exploitable for AML treatment.

## 7. Target-Based Therapy Using Clinically Available Agents 

### 7.1. Glutamine Metabolism Modulators/Inhibitors 

Modulators of glutamine metabolism are emerging for use in “glutamine-addicted” cancers including acute lymphoblastic leukemia (ALL) and AML. Asparaginase, an enzyme that hydrolyzes circulating asparagine and glutamine to aspartate and glutamate, respectively, is well-established for the treatment of ALL [29]. Recently, it has been reported that asparaginase-induced glutamine depletion can be used safely and effectively for treatment of AML [52,53]. Clinically available asparaginases are derived from two bacterial sources (*Escherichia coli* and *Erwinia chrysanthemi*), and while both have the dual asparaginase and glutaminase activity, asparaginase derived from *Erwinia*, called crisantaspase, has been shown to have a higher glutaminase activity. The use of L-asparaginase [L-Asp] is a well-established treatment in pediatric and adult ALL treatment [52,53,54]. PEGylated *E*. *coli* L-Asp is an asparaginase that underwent the process of covalent and non-covalent attachment of polyethylene glycol. PEG L-Asp has a longer half-life and is an FDA-approved drug for ALL therapy. PEGylated *Erwinia Chrysantemi*, PegCrisantaspase (PegC), a long-acting asparaginase, is under clinical and preclinical investigation for ALL and AML [52]. In addition to glutamine depletion using an asparaginase, there are three other ways to target glutamine metabolism efficiently. Additional methods include the inhibition of glutamine transporters, inhibition of glutaminase or antagonizing glutamine directly (Table 1).

Gregory et al. [42] showed that when glutamine metabolism is restricted or hindered by a small molecule glutaminase inhibitor (CB-839) in a FLT-3 mutated AML in vitro model, cellular proliferation, mitochondrial respiration, and GSH production are all negatively affected. Moreover, their patient-derived xenograft model of FLT-3 mutated AML showed a decrease in leukemia burden following one week of CB-839 treatment. In a different study, Polet et al. [11] demonstrated that the expression of key enzymes involved in the serine biosynthesis pathway, specifically PHGDH and PSAT are increased following glutamine removal from tissue culture as well as glutamine depletion induced by L-Asp. These findings suggest that co-targeting serine and glutamine metabolisms may have synergistic activity against AML cells.

### 7.2. Serine Biosynthesis Modulators/Inhibitors 

Cancer cell lines that overexpress the rate-limiting enzyme involved in the serine biosynthesis pathway PHGDH are sensitive to the pharmacologic inhibition of PHGDH, suggesting that targeting this enzyme may have a therapeutic role [11,55]. One approach to target PHGDH is to use the small molecule inhibitor CBR-5884 [6]. This molecule specifically blocks the serine biosynthesis pathway without affecting other glycolytic intermediates. In their study, Mullkary et al. [6] showed significant decrease in cellular proliferation and growth in breast cancer and melanoma in vitro models following the inhibition of PHGDH by CBR-5884. 

NCT-503, another PHGDH inhibitor, is under pre-clinical investigation in multiple myeloma. Elsaadi et al. [56] showed that myeloma cells treated with NCT-503 had reduced intracellular redox capacity and that inhibition of PHGDH provided a therapeutic advantage in vivo when combined with proteasome inhibitor bortezomib. These studies further advance the concept of using a small-molecule inhibitor for PHGDH as an option for cancers that overexpress this enzyme, which includes AML [10,57]. These findings suggest small molecule inhibitors of PHGDH are worth investigating in an AML model.

### 7.3. Anti-Folates and Anti-Metabolites 

The use of anti-metabolites, including anti-folates, has shown to be successful for the treatment of patients with lymphoid malignancies including ALL and chronic lymphocytic leukemia (CLL) as well as gastrointestinal (GI) malignancies including pancreatic and colorectal adenocarcinomas [22]. The mechanism of action of anti-folates is through the inhibition of key enzymes that are involved in the folate pathway [58,59]. These enzymes include (1) thymidylate synthase (TS), an enzyme responsible for the methylation of deoxyuridine monophosphate (dUMP) to deoxythymidine monophosphate (dTMP) in order to incorporate thymidine into DNA [4], (2) dihydrofolate reductase (DHFR), an enzyme that reduces dihydrofolate to tetrahydrofolate which can be converted to tetrahydrofolate cofactors [60], and (3) glycinamide ribonucleotide formyltransferase (GARFT), which is involved in the pathway that produces inosine monophosphate (IMP), a precursor to adenosine monophosphate (AMP) and guanosine monophosphate (GMP), two important building blocks for energy-carrying molecules such as adenosine triphosphate (ATP) [61]. 

In addition to anti-folates, the anti-metabolites 5-fluorouracil (5-FU) and capecitabine are widely used in many epithelial tumors particularly upper and lower GI cancers. 5-FU is a pyrimidine analog and a potent inhibitor of TS, which causes disruption and cessation of the folate cycle [18]. Capecitabine is a prodrug of 5-FU that is metabolized to two active metabolites: 5-fluorouridine triphosphate (FUTP) and 5-fluoro-2-deoxyuridine monophosphate (FdUMP). Of the two metabolites FUTP inhibits RNA and protein synthesis and FdUMP inhibits DNA synthesis. The use of anti-folates and anti-metabolites disrupt 1-C metabolism which then, in turn, disrupts DNA and RNA synthesis preventing cancer cells from proliferation [18,22]. These drugs have not been studied in combination with glutamine depletion, which makes our proposal novel. 

## 8. Conclusions

Chemotherapeutics are widely used as the first line of treatment for AML in young and medically fit patients; however, despite the new FDA approvals for AML and expanded availability and use of allogeneic stem cell transplantation, the five year survival rate for patients with AML remains less than 40% [1]. In this review, we discussed multiple components of 1-C cellular metabolism, including the serine, folate, and methionine pathways, and how they are modulated in neoplastic cells, as well as how they can be exploited for the treatment of AML and potentially other cancers.

In addition to targeting these pathways with known clinically available FDA approved drugs, such as anti-folates and other anti-metabolites, we are proposing to add glutamine-targeting agents as a novel therapeutic approach due to the interplay between 1-C metabolism and glutamine metabolism. Given the evidence of interaction between these metabolic pathways discussed in this review, our co-targeting approach is worth investigating. Several drugs targeting these pathways are already clinically available; hence, there is the potential for a rapid bench to bedside transition to clinically test these strategies against AML. 

In addition to potentially providing new treatments, exploiting these pathways can result in the development of prognostic and predictive biomarkers such as measuring/monitoring metabolites in biological fluids including bone marrow aspirate, plasma and urine [11,24].

## Figures and Tables

**Table 1:** Drugs Targeting Glutamine, Serine, and 1-C Metabolism.

Classification	Mechanism of Action	Examples	Pre-Clinial/Clinial Usage
**Glutamine Metabolism**			
Asparaginase	Depletes glutamine in the plasma by converting glutamine/asparagine to glutamate/aspartate and ammonia	*E.Coli* [Asparaginase (short acting), pegasparagase (long acting) *Erwinia Chrysantemi* [Erwinaze or crisantasapase (short acting)]; PegCrisantaspase [(PegC), long acting, under clinical and pre-clinical investigation for ALL and AML]	ALL [40] Pre-clinical studies in AML [11,30,52,54]
Glutaminase Inhibitor	Inhibits the hydrolase enzyme that converts glutamine to glutamate	BPTES CB-839	Pre-clinical studies in Breast Cancer [41] and AML [42]
Glutamine Transporter Inhibitor	Inhibits transport of glutamine in the cell (PM and mitochondria)	V-9302	Pre-clinical studies in CRC [43]
Glutamine Antagonist/Analog	Direct blockage of glutamine metabolism	JHU083 L-DON	Pre-clinical studies in Medulla Blastoma [45]
**Serine Biosynthesis**			
Phosphoglycerate dehydrogenase (PHGDH) Inhibitor	Inhibits the rate limiting step of serine biosynthesis (PHGDH)	CBR-5884 NCT-503	Pre-clinical studies in Breast Cancer [6,44] and AML [10]
Serine hydroxymethyltransferase (SHMT) Inhibitor	Inhibits the enzyme (SHMT) that catalyzes the conversion of serine to glycine and initiates the folate cycle	SHIN2	Pre-clinical studies in T-ALL [16]
**1-C Metabolism**			
Anti-Folates	Antagonize the actions of folic acid and inhibit key enzymes in the folate cycle including: Thymidylate synthase (TS), Dihydrofolate reductase (DHFR), Glycinamide ribonucleotide formyltransferase (GARFT)	Methotrexate Pemetrexed	ALL, Breast Cancer, HNSCC, Non-Hodgkin Lymphoma and Osteosarcoma [21,22] Malignant pleural mesothelioma Non-squamous NSCL cancer
Anti-Metabolites	Analogues of a normal metabolites that inhibit key enzymes in the folate cycle including: Thymidylate synthase (TS)	5-Fluorouracil Capecitabine	Colorectal Cancer, Breast Cancer, Gastrointestinal Cancers and HNSCC [22]
